# The benefits of psychosocial interventions for mental health in men who have sex with men living with HIV: a systematic review and meta-analysis

**DOI:** 10.1186/s12888-022-04072-1

**Published:** 2022-06-29

**Authors:** Yan Yu, Xinyu Wang, Yaxin Wu, Wenjia Weng, Ming Zhang, Juan Li, Xiaojie Huang, Yanqing Gao

**Affiliations:** 1 Department of Dermatology, Beijing Youan Hospital, Capital Medical University, Beijing, China; 2 Center for Infectious Diseases, Beijing Youan Hospital, Capital Medical University, Beijing, China; 3 Candidate Branch of National Clinical Research Center for Skin Diseases, Beijing, China

**Keywords:** HIV, MSM, Psychosocial intervention, Mental health, Meta-analysis

## Abstract

**Objective:**

Men who have sex with men (MSM) living with HIV are more likely to suffer from mental health problems. They should be given adequate attention to treat and improve their mental health disorders. This meta-analysis aimed to assess whether psychosocial interventions reliably improve psychological well-being among MSM living with HIV.

**Method:**

Cochrane Library, EMBASE, PsycINFO, and PubMed were searched for psychosocial intervention randomized controlled trials evaluating mental health (e.g., depression, anxiety, self-efficacy). The effect size was pooled using the random-effects model, and continuous outcomes were reported using standardized mean difference (*SMD*) values .

**Results:**

A total of 12 studies including 1782 participants were included in the meta-analysis. Psychosocial interventions in contrast to control groups significantly reduced depression (*SMD*, − 0.28; 95% CI − 0.52 – − 0.03) at the follow-up assessment and improved quality of life (*SMD* 0.43, 95% CI 0.23–0.63) after treatment. Psychosocial interventions also had a significant effect on measures of self-efficacy (*SMD* 2.22, 95% CI 0.24–4.20), and this effect was sustained until long-term follow-up (*SMD* 0.55, 95% CI 0.02–1.08). Subgroup analyses revealed that improvements in depression were more significant when participants possessed higher education and treatment providers used cognitive behavioral therapy (CBT).

**Conclusions:**

The findings of this study indicate that psychosocial interventions benefit the mental health of MSM living with HIV. It is necessary to conduct more research to explore characteristics that may affect treatment outcomes in the future.

**Trial registration:**

This research was prospectively registered in PROSPERO (CRD42021262567).

**Supplementary Information:**

The online version contains supplementary material available at 10.1186/s12888-022-04072-1.

## Background

Men who have sex with men (MSM) are susceptible to HIV infection. According to the US Centers for Disease Control and Prevention, the HIV infection rate among this population is 19%, with about half of new HIV infections occuring among them [1, 2]. Double pressures of HIV-related stigma and sexual minority stress pose a threat to the mental health of MSM living with HIV, which may lead to depression, anxiety, stress, and poor coping skills [3–5]. MSM living with HIV have a relatively high prevalence of depression, especially with the interaction of HIV-induced neuroendocrine, immune-inflammatory and monoaminergic mechanisms and psychosocial factors [6]. For example, a recent meta-analysis showed that 43% of MSM living with HIV experienced depression [2]. Mental health problems may have adverse consequences for MSM living with HIV, and are a huge obstacle to their participation in nursing, HIV testing, as well as initiating and adhering to antiretroviral therapy (ART) [7, 8].

Considering the impact of psychological comorbidities on the health and mental wellbeing of MSM living with HIV, interventions are imperative. In recent decades, psychosocial therapy has developed rapidly because of its flexibility, relatively low cost and limited side effects compared with pharmacotherapy [9]. Psychosocial interventions have been widely used to address mental disorders in MSM living with HIV. Such interventions mainly focus on psychological or social factors rather than solely on exercise or pharmacological treatment and include interventions such as cognitive behavioral therapy (CBT), relaxation, stress-management, motivational interviewing, coping effectiveness training, support techniques and mindfulness.

A systematic review and meta-analysis of seven studies showed that CBT had a short-term effect on depressive symptoms in people living with HIV (PLWH) with depression [10]. In addition, systematic reviews and meta-analyses involving various psychological interventions have demonstrated their benefits in improving the psychological well-being of mixed populations [11–14]. Pantalone [15] conducted a meta-analysis of combined behavioral interventions that jointly targeted HIV-related health behaviors and psychosocial symptoms, showing a significant small but positive effect in improving the mental health of sexual minority men.

Thus far, there has been no meta-analysis that examines the impact of psychosocial intervention on the mental health of MSM living with HIV. As trials of psychosocial interventions and research interest in MSM living with HIV are carried out, there is a need to summarize relevant studies to provide better treatment for this minority population. This meta-analysis assessed the summarized effects of psychosocial interventions on depression, anxiety, stress, quality of life, and self-efficacy among MSM living with HIV, and investigated the moderators of the intervention effect to provide more targeted interventions.

## Methods

### Protocol and registration

This meta-analysis is reported in accordance with the Preferred Reporting Items for Systematic Reviews and Meta-Analyses (PRISMA) [16]. The PRISMA checklist for the study is shown in Additional file 1 (supplementary material). This meta-analysis was prospectively registered in PROSPERO (CRD42021262567).

### Inclusion criteria

The general criteria for the included studies were as follows: (1) MSM living with HIV, aged 18 years and older; (2) the study contained depression, anxiety, stress, quality of life, social support, or self-efficacy as a primary or secondary outcome; (3) only pilot RCT were included; and (4) the study employed a psychosocial intervention aimed to improve the psychological health of MSM infected with HIV.

### Exclusion criteria

The exclusion criteria were as follows: (1) case series, literature reviews, or study protocols; (2) studies that did not offer sufficient information for meta-analysis; and (3) studies written in non-English languages. If multiple papers were published based on the same data, only the most relevant article to the outcome data was included.

### Literature search strategies

Four databases (Cochrane Library, EMBASE, PsycINFO, and PubMed) were searched, with dates ranging from January 1, 1996 to May 31, 2021. The following search terms were used: HIV infection, HIV, acquired immune deficiency syndrome, AIDS, MSM, gay, and psychosocial intervention-related words. The search strategy using keywords is provided in Additional file 2. To include more studies, references to related articles were manually searched. Two review authors (Y.Y. and X.W.) independently screened articles for selection in the study.

### Data extraction

Two reviewers (Y.Y. and X.W.) used a standardized data extraction table to obtain data from studies that fulfilled the criteria independently. The extracted data included study details (authors, year of publication, location), sample information, intervention and control group format, and relevant outcomes with measures (scales), etc.

### Risk of bias assessment

Two authors (Y.Y. and X.W.) independently assessed each study using the Cochrane Collaboration Risk of Bias tool [17]. The tool has seven domains: random sequence generation, allocation concealment, performance bias, detection bias, attrition bias, reporting bias, and other biases. Studies were rated with a low, high, or unclear risk of bias in each domain. Reviewers judged a study as “good quality” when the risk of judgment bias of all seven standards was low, “general quality” when the risk of judgment bias of one of the seven standards was high, or “poor quality” when the risk of judgment bias of two or more criteria were high. Studies with a low risk of bias meeting at least two criteria of the Cochrane bias risk tool were selected for meta-analysis. When the two authors did not resolve any disagreement after discussion, we consulted the third author (W.W.). In addition, we attempted to contact the study authors for clarification.

### Treatment effect analysis

The Stata 16.0 software was used for the analysis. When the included studies evaluate the same outcome, but with different measurement methods or different scales, standardized mean difference (*SMD*) was considered suitable for selection as the pooled statistic to report continuous outcomes [18]. Means, standard deviations (*SD*), and sample sizes were used to calculate the effect sizes. We calculated Hedges’ g to represent the effect size. We used Cohen’s guidelines to describe the effect size: 0.2, 0.5, and 0.8 indicated small, medium, and large effect sizes, respectively [19]. All analyses used two-tailed *p*-values. We used random effect analysis because of clinical heterogeneity [20]. We assessed statistical heterogeneity based on Cochran’s Q statistic, where *p* < 0.10 implied significant heterogeneity. The *I*^*2*^ statistical values of 0–25%, 26–50%, and above 50% indicates low heterogeneity, medium heterogeneity, and high heterogeneity, respectively. Since we only included randomized controlled trials, we assumed that the intervention and control groups had no significant differences in mental health at baseline. We analyzed each result (i.e., depression, anxiety, stress, quality of life, social support, and self-efficacy) separately and stratified the primary analysis by time points: at the end of the intervention and at the longest follow-up. Probable reasons for statistical heterogeneity were explored through the following subgroup analyses: mean age, education, employment, frequency of session, provider of intervention, etc. The Egger test [21] and funnel plot by *SMD* [22] evaluated possible publication bias.

## Results

### Study selection

**Fig. 1:**
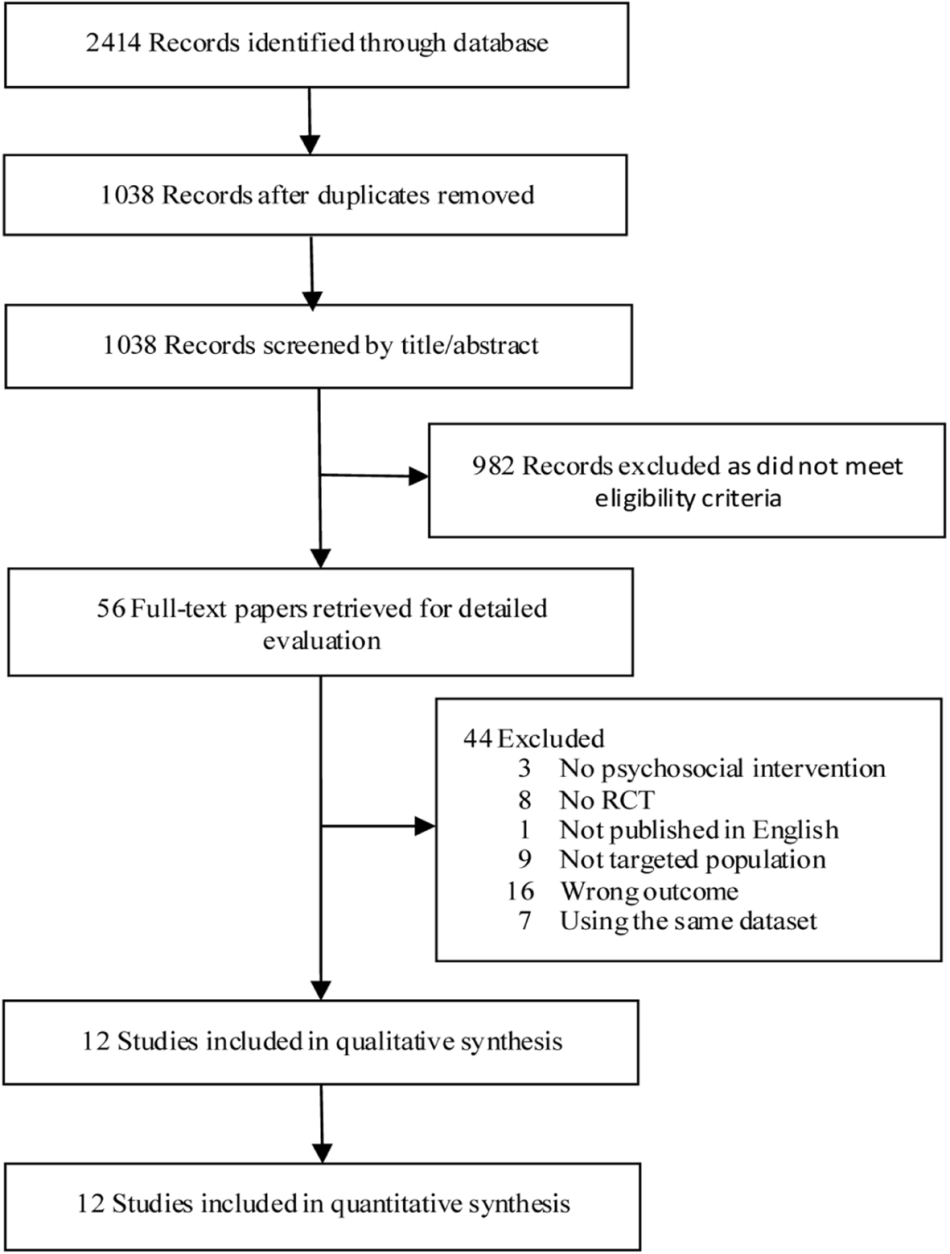
PRISMA flow diagram

We identified 2414 records from the electronic database searches. After eliminating 1376 copies, 1038 articles were selected based on the title and abstract. We screened the full texts of 56 articles. Ultimately, 12 studies met the inclusion criteria. The selection process for the meta-analysis is shown in Fig. 1.

### Characteristics of the included studies

The key characteristics of the included studies from the United States [23–28], the Netherlands and Belgium [29], Australia [30], Canada [31], Thailand [32], and China [33, 34] are presented in Table 1. A total of 1782 MSM living with HIV were included in the 12 studies. The sample size ranged from 40 [32] to 420 [33]. The average age of the study participants ranged from 29.3 [32] to 46.8 [24] years. Two studies cited additional participant inclusion criteria. One study recruited only those who had lost a friend or partner to AIDS in the past 6 months [28], and another recruited only those who had body image disturbance [24]. All studies excluded any active major mental disorders, such as cognitive impairment, psychosis, or major depression with active suicidality. Recruitment was conducted in clinics, hospitals, HIV/AIDS service organizations, and community settings.

**Table 1:** Basic characteristics of the studies included in the meta-analysis (*N* = 12)

Author (Year)	Country	Sample, N (%)	Age at baseline, ***Means*** ± ***SD***, y	University or above, N (%)	Employed, N (%)
Antoni et al. (2006) [23]	USA	130 (7.3)	41.6 ± 8.6	95 (73.1)	44 (33.8)
Carrico et al. (2005) [26]	USA	129 (7.2)	35.6 ± 7.2	107 (82.9)	101 (78.3)
Blashill et al. (2017) [24]	USA	44 (2.5)	46.18 ± 11.03	34 (77.3)	20 (45.5)
Brown et al. (2019) [25]	USA	79 (4.4)	40.6 ± 8.0	NR	39 (49.4)
Chesney et al. (2003) [27]	USA	105 (5.9)	39.0 ± 7.3	65 (61.9)	NR
Goodkin et al. (1999) [28]	USA	97 (5.4)	36.5 ± 7.7	NR	86 (88.5)
Weiss et al. (2003) [29]	Netherlands and Belgium	85 (4.8)	39	11 (12.9)	59 (69.4)
Millard et al. (2016) [30]	Australia	132 (7.4)	42.3 ± 10.4	95 (72.0)	93 (70.5)
Gayner et al. (2012) [31]	Canada	117 (6.6)	44	91 (77.8)	NR
Khumsaen et al. (2019) [32]	Thailand	40 (2.2)	29.30 ± 7.06	16 (40.0)	30 (75.0)
Zhang et al. (2019) [33]	China	420 (23.6)	NR	272 (64.8)	NR
Li et al. (2021) [34]	China	404 (22.7)	NR	162 (40.1)	NR

*Note*. *NR* Not reported in paper

Depression was measured in seven studies [23, 24, 26–29, 31], anxiety in four [27–29, 31], stress in three [25, 27, 31], quality of life in two [32, 33], social support in seven [25–27, 29, 30, 32, 34], and self-efficacy in three studies [25, 27, 30]. Regarding the type of control group, six studies used the treatment-as-usual (TAU) condition, that is treatment and care according to guidelines or general practice in primary or secondary care [24, 28, 30–33], two studies used the waiting list control condition [25, 26], and four studies used placebo control condition, including medication adherence training for participants aimed at increasing knowledge about HIV and ART [23], providing information on HIV-related topics and resources such as clinical trials, general health, disability and legal issues [27, 29], and weekly mental health promotion messages were sent to participants via social networks without any interaction [34]. Ten studies included one or more follow-up assessments [23–27, 29–31, 33, 34]. The follow-up time ranged from 3 [25] to 15 months [23, 29] after interventions.

Regarding techniques used in interventions, CBT techniques were used in six interventions [23–26, 33, 34], relaxation techniques in four [23, 26, 27, 29], stress-management techniques in six [23, 25–29], motivational interviewing techniques in four [23–26], coping effectiveness trainings in four [27–29, 34], support techniques in four [28–30, 34] and mindfulness techniques in two interventions [24, 31]. Only two studies involved online interventions [30, 34]. The remaining studies were face-to-face interventions [23–29, 31–33]. Providers of the face-to-face interventions were specialists (e.g., psychologists and/or psychotherapists) [24, 28, 29, 31, 33] or non-specialists (e.g., peers) [23, 25–27, 32]. Ten interventions [23, 25–31, 33, 34] used a group approach, one [24] used an individual approach, and the other [32] used a combination of individual and group approaches**.** After excluding the study using social networking (brief messages were posted to the group every day), the number of sessions ranged from 2 [25] to17 [29], with a mean of 10. One session lasted from 50 [24] to 240 [25] minutes. The average length of one session was 130 minutes, with a median of 135 minutes. For more details, see Additional file 3.

### Quality of included studies

For a summary of the “Risk of bias” findings, see Additional file 4. There were five studies at low risk of selection bias to provide sufficient details to support their judgements [23, 24, 30, 31, 34]. Participants had difficulty being blinded to the allocation to the conditions in almost all studies. The majority did not mention allocation concealment or detection bias. Drop-out rates ranged from 0% [28, 32] to 66% [26] and only three studies [24, 28, 32] were evaluated as having a low risk of attrition bias. Intention-to-treat (ITT) analysis was performed in eight trials [23–26, 29–31, 33]. Only one trial [30] published the protocol. No evidence of selective reporting was found, and all included studies were evaluated as having a low risk of reporting bias. We surmised that all included studies had a low risk of other biases because there was no imbalance in potential confounding factors between the intervention and control groups at baseline.

### Meta-analysis and effect size

#### The effect of psychosocial intervention on depression

**Fig. 2:**
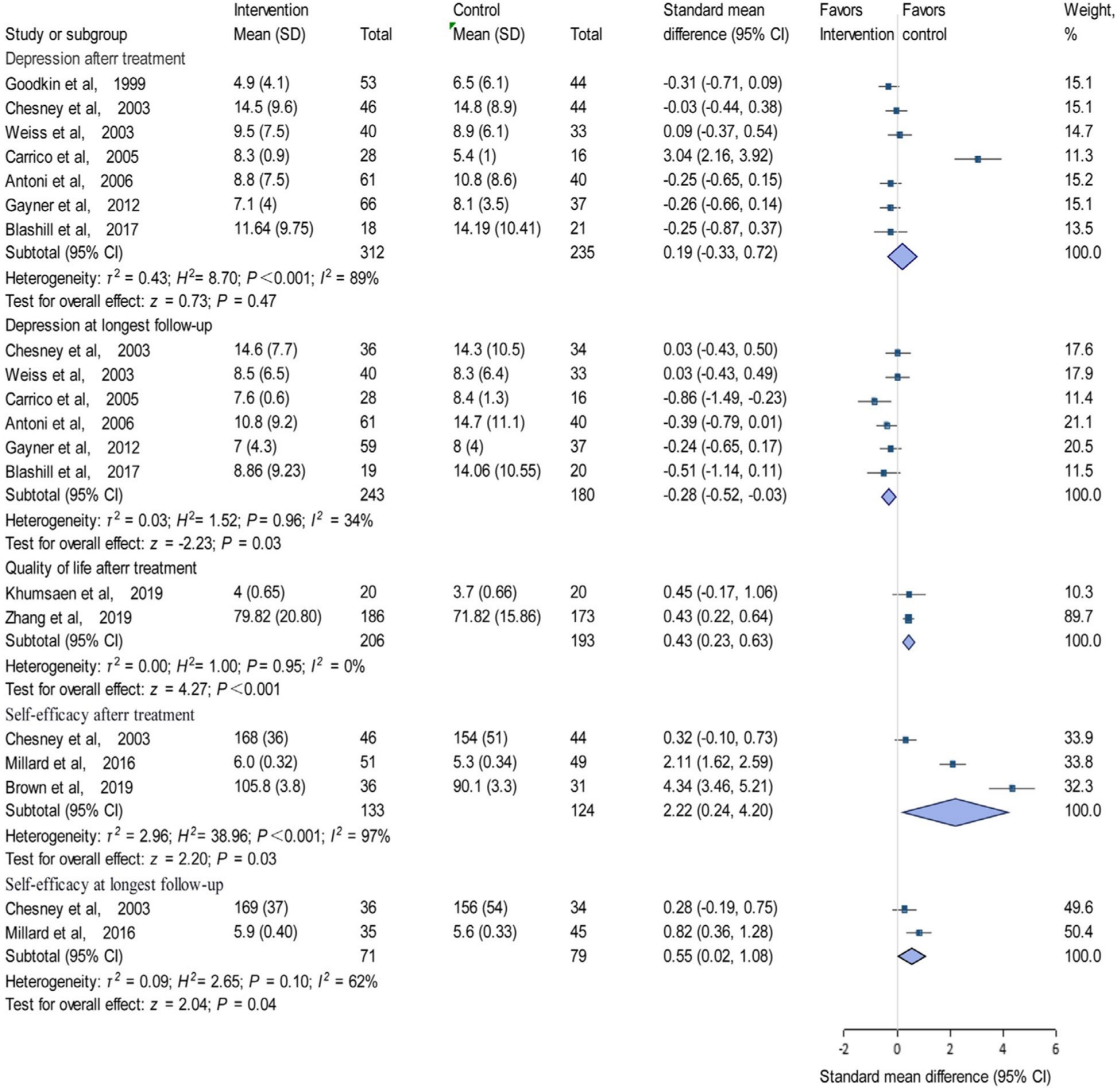
Forest plot of effect sizes for measures of depression, quality of life and self-efficacy

The average depression score of the intervention group did not decrease significantly compared with the control group after treatment (*SMD*, 0.19; 95% CI, − 0.33–0.72; *P* > 0.05; Fig. 2). To observe long-term outcomes, we analyzed the effect sizes at the last follow-up time point. Notably, for depression scores at the last follow-up assessment, the meta-analysis showed a significant effect size (*SMD*, − 0.28; 95% CI, − 0.52−− 0.03; *P* < 0.05; Fig. 2).

#### The effect of psychosocial intervention on anxiety

There was no significant effect of anxiety at the endpoint of treatment (SMD, − 0.19; 95% CI, − 0.40–0.01; *P* > 0.05; Additional file 5), or at the endpoint of follow-up (SMD, − 0.11; 95% CI, − 0.36–0.15; *P* > 0.05; Additional file 5).

#### The effect of psychosocial intervention on stress

For stress, the meta-analysis showed no significant effect after intervention (*SMD*, − 1.16; 95% CI, − 2.63–0.32; *P* > 0.05; Additional file 5), or at the follow-up assessment (*SMD*, − 0.29; 95% CI, − 0.61–0.03; *P* > 0.05; Additional file 5).

#### The effect of psychosocial intervention on quality of life

Regarding quality of life, the meta-analysis showed a significant effect size (*SMD*, 0.43; 95% CI, 0.23–0.63; *P <* 0.001; Fig. 2) based on two trials with 399 participants after treatment. Two studies did not provide enough information to calculate the effect size at long-term follow-up.

#### The effect of psychosocial intervention on social support

There was no significant effect for social support at the post-treatment assessment (*SMD*, 0.15; 95% CI, − 0.49–0.79; *P* > 0.05; Additional file 5), or at the endpoint of follow-up (*SMD*, 0.4; 95% CI, − 0.04–0.85; *P* > 0.05; Additional file 5).

#### The effect of psychosocial intervention on self-efficacy

Compared with the control group, psychosocial interventions positively affected self-efficacy. The effect size was large (*SMD*, 2.22; 95% CI, 0.24–4.20; *P* < 0.05; Fig. 2) at the end of the intervention. Moreover, the effect was reduced, but still significant, at long-term follow-up (*SMD*, 0.55; 95% CI, 0.02–1.08; *P* < 0.05; Fig. 2).

#### Subgroup analyses

As shown in Table 2, we conducted a series of subgroup analyses on the outcomes of depression. Education was a significant moderator; that is, the effect sizes were greater in the studies with at least 70% of participants with a college degree or higher. In addition, In addition, interventions that used CBT techniques were more effective than those that did not.

**Table 2:** Subgroup analyses of psychosocial interventions for depression

Subgroups	Studies, No.	Sample, No.	***SMD***	95% CI	***I***^***2***^, %	***P*** value for interaction
Mean age						
<40 years	3	187	− 0.22	[− 0.73, 0.29]	66	0.67
>40 years	3	236	−0.35	[− 0.61, − 0.08]	0	
Control group						
TAU	2	135	−0.32	[− 0.66, 0.03]	0	0.12
Waiting list	1	44	−0.86	[−1.50, − 0.22]	NA^a^	
Placebo	3	244	−0.13	[− 0.42, 0.16]	20	
%University or above						
<70	2	143	0.03	[−0.30, 0.36]	0	0.03^b^
>70	4	280	−0.42	[− 0.66, − 0.18]	0	
%Employment						
<50	2	117	−0.42	[− 0.76, − 0.08]	0	0.94
>50	2	140	−0.39	[−1.26, −0.49]	80	
Frequency of session						
≤ 10 sessions	3	210	−0.31	[−0.75, 0.14]	59	0.88
>10 sessions	3	213	−0.26	[− 0.58, 0.05]	20	
Provider of intervention						
Specialist	3	208	−0.19	[− 0.47, 0.08]	0	0.52
Non-specialist	3	215	−0.37	[−0.83, 0.09]	61	
Form of intervention						
Individual	1	39	−0.51	[−1.15, 0.13]	NA^a^	0.45
Group	5	384	−0.25	[− 0.52,0.02]	40	
Duration of one session						
<2 h	2	109	−0.20	[− 0.72, 0.33]	45	0.70
>2 h	4	314	−0.32	[− 0.62, − 0.01]	42	
Cognitive behavioral technique						
Yes	3	184	−0.52	[− 0.82, − 0.22]	0	0.03^b^
No	3	239	−0.07	[−0.33, 0.18]	0	
Length of follow-up						
<12 months	3	179	−0.47	[− 0.83, − 0.11]	24	0.15
≥ 12 months	3	244	−0.13	[−0.42, 0.16]	20	

*Note*. *NA* not applicable, *SMD* standardized mean difference

^a^I^2^ is not calculated here because only 1 study reported this outcome

^b^*p* < 0.05

#### Publication bias assessment

**Fig. 3:**
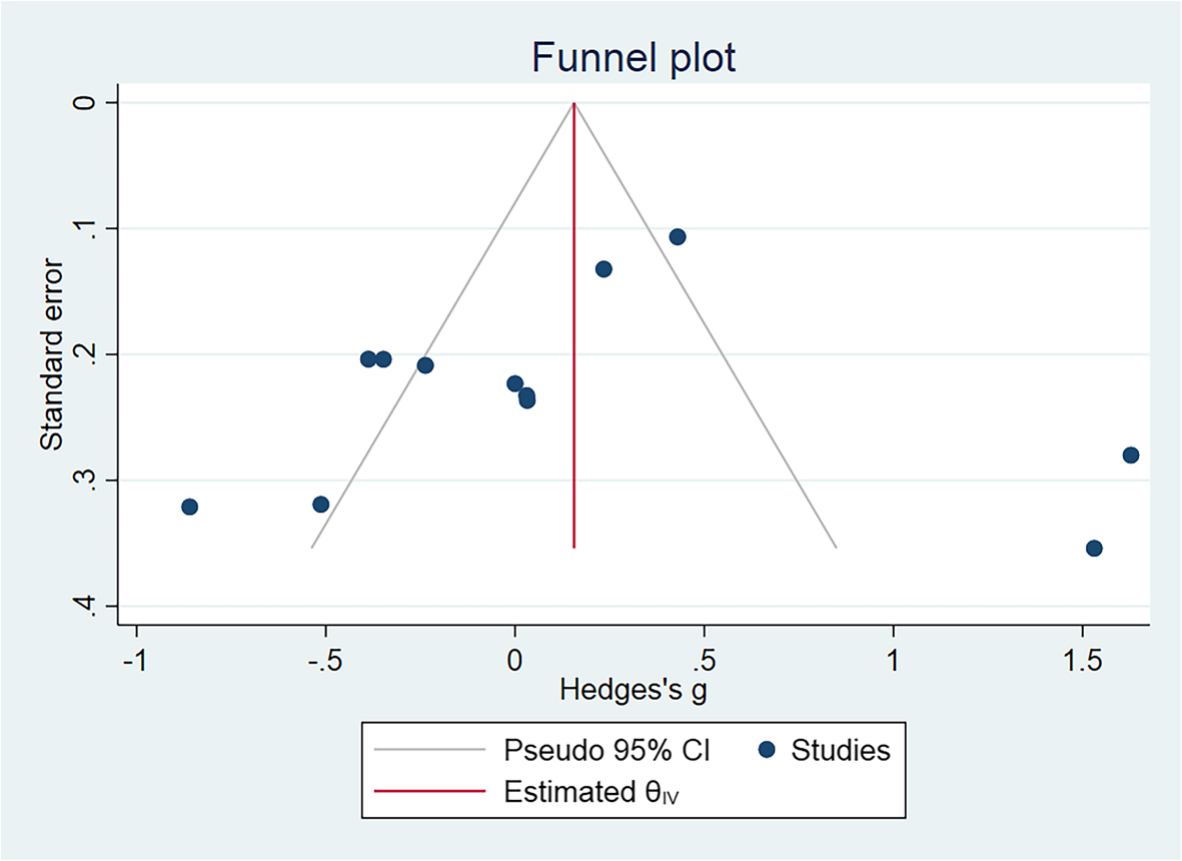
Funnel plot of standard error by Hedges’s g

In general, the included studies showed a symmetrical distribution in the funnel chart (Fig. 3). Egger’s test also indicated no obvious publication bias (*P* = 0.65).

## Discussion

This is the first systematic review and meta-analysis to examine the efficacy of psychosocial interventions for MSM infected with HIV on mental health. We identified the essential benefits of psychosocial treatment for self-efficacy, quality of life, and depression. We found that the sizes of the pooled effect on self-efficacy were the largest after interventions, and this effect was sustained until the last follow-up after participation in group sessions. MSM living with HIV always face social context vulnerabilities that reduce their self-efficacy and delay HIV self-management behaviors. This includes good adherence to antiretroviral therapy, engaging in protected sex, and cultivating self-regulatory behaviors [35, 36]. The self-management program is based on self-efficacy and self-management theories, combined with a variety of psychosocial intervention techniques such as relaxation, support, and exercise, which could enhance confidence, improve self-efficacy, and facilitate development of good habits. Several included studies emphasized participants’ self-management [25, 27, 30], which may also explain the large self-efficacy effect. To coexist with HIV infection, MSM living with HIV learning how to manage their chronic disease may be a trend in future treatment.

In addition, our review reported a smaller effect on depression and quality of life, which was identical to earlier meta-analysis results [12]. Depression is a robust predictor of social isolation, high suicidal ideation, low adherence to ART, and poor viral suppression [37]. The significance of depression goes beyond the number of studies that have been conducted or that met inclusion criteria. Although psychosocial intervention did not immediately reduce depression in MSM living with HIV after treatment, its benefits could be observed during the long-term follow-up. For future research, it is crucial to expand the sample size and monitor participants over an extended period to provide more evidence.

There were no differences in anxiety, social support, or stress between the groups. Similarly, a recent meta-analysis showed that psychosocial interventions based on mindfulness or CBT may not significantly affect levels of anxiety and stress [11]. More restrictions on the disclosure of weaknesses or negative views by MSM, who are a sexual minority, may prevent them from perceiving and receiving higher levels of support. Since most trials focused on depression, fewer systematic reviews and meta-analyses have included other psychological outcomes. This suggests that we should be cautious in interpreting the results and pay appropriate attention to other psychological problems in the future.

Our subgroup analysis showed that several characteristics impacted the effectiveness of the treatment for depression. The subgroup analyses demonstrated that participants with higher education benefitted more from interventions. This may be because those who are more highly educated are better able to make use of these interventions [38–40]. In addition, people with higher levels of education also have higher socioeconomic status in general, which enables better self-protection from the vulnerabilities of poor mental health.

We found that the effect of using CBT technology was greater than interventions that did not use CBT technology. CBT is a short-term psychotherapy method that changes cognition by eliminating destructive emotions and behaviors and is widely used to treat a variety of mental illnesses [41, 42]. A meta-analysis has shown that CBT can significantly improve depression in PLWH [10]. Considering the general efficacy of CBT, it can be applied during psychosocial interventions to reduce depression in MSM living with HIV.

The subgroup analysis also demonstrated no difference in the effects between studies where specialists (e.g., psychologists or psychiatrists) provided treatments, and those where well-trained research staff (e.g., psychology students) administered treatments. The results showed no difference from previous reviews [43–45]. A meta-analysis conducted in low- and middle-income countries also found that interventions conducted by inexperienced staff for the psychological disorders of PLWH were as effective as interventions administered by experienced professionals [46]. It is pertinent to use trained counselors and adopt a task-sharing approach to address the shortage of human resources in resource-limited settings. Furthermore, technology-based psychotherapeutic interventions have developed rapidly and are increasingly being used for PLWH, which has benefitted those with depression in recent years [47–50]. Given its effectiveness, low cost, and ease of communication, psychosocial intervention could play a more crucial role through online materials and telecommunication devices.

Most articles included in this review used group therapy, possibly because of its broader social and psychological benefits. Group therapy is a process of multidirectional communication, in which participants can learn the adaptive behavior of multiple group members, and glean insight into themselves from multiple perspectives [51]. Members support each other and jointly explore solutions to problems, which is especially suitable for people with maladaptive interpersonal relationships [52]. In addition, the group therapy mode has high consultation efficiency. One therapist instructs multiple patients simultaneously, which saves time and human resources, and is therefore cost effective and resource efficient. The subgroup analysis of this meta-analysis found no statistical difference in efficacy between group treatment and individual treatment, which is consistent with the results of Van Luenen et al. [12] and Asrat et al. [46]. A meta-analysis of technology-delivered psychotherapy for the intervention of depressive symptoms in HIV/AIDS patients showed that the individual approach had a larger effect size than the group approach. This may be because the individual approach could improve interpersonal connections within the HIV care continuum by providing customized counseling sessions [13]. The existence of heterogeneity and the small number of studies indicate the need for further research in this area by combining issues of cost, confidentiality and acceptability to compare and select appropriate intervention forms.

Moreover, we found no differences in effect between the duration of the intervention per session and the frequency of sessions, which is consistent with previous meta-analyses results regarding psychosocial interventions for anxiety or depression [44, 45, 53, 54]. One study indicated that concise treatments are a potentially viable alternative to standard mental health care for depression or anxiety [55]. A meta-analysis involving more studies suggested that treatments with a duration of 12–18 hours may be more effective than shorter or longer treatments [12]. Therefore, the relationship between the duration and effectiveness of the intervention remains unclear. Further research is necessary to determine whether the intervention process can be simplified, and the duration of treatment may thus be shortened to conserve resources.

At present, the burden of mental illness for PLWH is severely underestimated. The continued growth of health care and mental health expenditure poses a serious challenge for countries worldwide. As patients’ needs for mental health care increase, incorporating evidence-based positive psychosocial interventions such as CBT into established models of care for people living with HIV can be an innovative and cost-effective approach. Trained non-specialists can improve the mental health of MSM living with HIV through psychosocial interventions, in groups or through online or telemedicine, to alleviate shortages of funds and professionals in resource-poor settings and build well-being for this population.

### Limitations

Our meta-analysis has several limitations. First, although the results were evaluated using validated standard scales with good reliability and validity, variation in the tools used in different studies is a limitation of this meta-analysis. In addition, as there is heterogeneity among studies, interpretations of meta-analysis results should be made with caution. Second, most of the studies included in the meta-analysis were of fair quality, and only 25% of the included studies were of good quality. Third, fewer studies were eligible for the meta-analysis, there were insufficient studies to identify additional moderators, and some results had low degrees of freedom, leading to uncertainty in statistical inferences. Fourth, psychosocial interventions targeting clinical indicators, HIV-related health behaviors, and mental health-targeting are often isolated intervention development approaches, given the organization of the field and reducing confounding factors. Therefore, this meta-analysis could not explore the effects of psychosocial interventions on treatment compliance, HIV viral load, and high-risk sexual behavior, for example. Moreover, we only included studies published in English, which may have precluded research published in other languages.

## Conclusion

A systematic review and meta-analysis found that psychosocial interventions positively affect self-efficacy, quality of life, and depression in MSM living with HIV. Improvements in depression may be more significant among populations with higher education, and when CBT is used. In summary, it is necessary to conduct psychosocial interventions among MSM living with HIV to promote mental health among this population.

## Supplementary Information

**Additional file 1. **PRISMA 2020 Checklist.

**Additional file 2. **Detailed search strategy.

**Additional file 3. **Detailed characteristics in selected studies.

**Additional file 4. **Quality of included studies.

**Additional file 5. **Forest plot of effect sizes for anxiety, stress and social support.

## Data Availability

All data analyzed during this study are included in the original studies’publications and this article.

## Abbreviations

AIDS: Acquired immune deficiency syndrome
ART: Antiretroviral therapy
CBT: Cognitive behavioral therapy
HIV: Human immunodeficiency virus
MSM: Men who have sex with men
NA: Not applicable
NR: Not reported in paper
PLWH: People living with HIV
PRISMA: Preferred Reporting Items for Systematic Reviews and Meta-Analyses
SMD: Standardized mean difference

## References

[1] Hall HI, Song R, Rhodes P, Prejean J, An Q, Lee LM (2008). Estimation of HIV incidence in the United States. JAMA.

[2] Xiao L, Qi H, Wang YY, Wang D, Wilkinson M, Hall BJ (2020). The prevalence of depression in men who have sex with men (MSM) living with HIV: a meta-analysis of comparative and epidemiological studies. Gen Hosp Psychiatry.

[3] Brown MJ, Serovich JM, Kimberly JA (2016). Depressive symptoms, substance use and partner violence victimization associated with HIV disclosure among men who have sex with men. AIDS Behav.

[4] Stahlman S, Grosso A, Ketende S, Sweitzer S, Mothopeng T, Taruberekera N (2015). Depression and social stigma among MSM in Lesotho: implications for HIV and sexually transmitted infection prevention. AIDS Behav.

[5] Rasoolinajad M, Abedinia N, Noorbala AA, Mohraz M, Badie BM, Hamad A (2018). Relationship among HIV-related stigma, mental health and quality of life for HIV-positive patients in Tehran. AIDS Behav.

[6] Nanni MG, Caruso R, Mitchell AJ, Meggiolaro E, Grassi L (2015). Depression in HIV infected patients: a review. Curr Psychiatry Rep.

[7] Lee C, Oliffe JL, Kelly MT, Ferlatte O (2017). Depression and Suicidality in gay men: implications for health care providers. Am J Mens Health.

[8] Batchelder AW, Safren S, Mitchell AD, Ivardic I, O'Cleirigh C (2017). Mental health in 2020 for men who have sex with men in the United States. Sex Health.

[9] Linden M, Schermuly-Haupt ML (2014). Definition, assessment and rate of psychotherapy side effects. World Psychiatry.

[10] Shi Y, Zhao M, Chen S, Wang S, Li H, Ying J (2019). Effects of cognitive behavioral therapy on people living with HIV and depression: a systematic review and meta-analysis. Psychol Health Med.

[11] van der Heijden I, Abrahams N, Sinclair D (2017). Psychosocial group interventions to improve psychological well-being in adults living with HIV. Cochrane Database Syst Rev.

[12] van Luenen S, Garnefski N, Spinhoven P, Spaan P, Dusseldorp E, Kraaij V (2018). The benefits of psychosocial interventions for mental health in people living with HIV: a systematic review and Meta-analysis. AIDS Behav.

[13] Cheng LJ, Kumar PA, Wong SN, Lau Y (2020). Technology-delivered psychotherapeutic interventions in improving depressive symptoms among people with HIV/AIDS: a systematic review and Meta-analysis of randomised controlled trials. AIDS Behav.

[14] Bhochhibhoya A, Harrison S, Yonce S, Friedman DB, Ghimire PS, Li X (2021). A systematic review of psychosocial interventions for older adults living with HIV. AIDS Care.

[15] Pantalone DW, Nelson KM, Batchelder AW, Chiu C, Gunn HA, Horvath KJ (2020). A systematic review and Meta-analysis of combination behavioral interventions co-targeting psychosocial Syndemics and HIV-related health behaviors for sexual minority men. J Sex Res.

[16] Moher D, Liberati A, Tetzlaff J, Altman DG (2009). Preferred reporting items for systematic reviews and meta-analyses: the PRISMA statement. Ann Intern Med.

[17] Higgins JP, Altman DG, Gøtzsche PC, Jüni P, Moher D, Oxman AD (2011). The Cochrane collaboration’s tool for assessing risk of bias in randomised trials. BMJ.

[18] Andrade C (2020). Mean difference, standardized mean difference (SMD), and their use in Meta-analysis: as simple as it gets. J Clin Psychiatry.

[19] Cohen J (1992). A power primer. Psychol Bull.

[20] Borenstein M, Hedges LV, Higgins JP, Rothstein HR (2010). A basic introduction to fixed-effect and random-effects models for meta-analysis. Res Synth Methods.

[21] Egger M, Davey Smith G, Schneider M, Minder C (1997). Bias in meta-analysis detected by a simple, graphical test. BMJ.

[22] Zwetsloot PP, Van Der Naald M, Sena ES, Howells DW, IntHout J, De Groot JA (2017). Standardized mean differences cause funnel plot distortion in publication bias assessments. Elife.

[23] Antoni MH, Carrico AW, Durán RE, Spitzer S, Penedo F, Ironson G (2006). Randomized clinical trial of cognitive behavioral stress management on human immunodeficiency virus viral load in gay men treated with highly active antiretroviral therapy. Psychosom Med.

[24] Blashill AJ, Safren SA, Wilhelm S, Jampel J, Taylor SW, O'Cleirigh C (2017). Cognitive behavioral therapy for body image and self-care (CBT-BISC) in sexual minority men living with HIV: a randomized controlled trial. Health Psychol.

[25] Brown JL, Vanable PA, Bostwick RA, Carey MP (2019). A pilot intervention trial to promote sexual health and stress management among HIV-infected men who have sex with men. AIDS Behav.

[26] Carrico AW, Antoni MH, Weaver KE, Lechner SC, Schneiderman N (2005). Cognitive-behavioural stress management with HIV-positive homosexual men: mechanisms of sustained reductions in depressive symptoms. Chronic Illn.

[27] Chesney MA, Chambers DB, Taylor JM, Johnson LM, Folkman S (2003). Coping effectiveness training for men living with HIV: results from a randomized clinical trial testing a group-based intervention. Psychosom Med.

[28] Goodkin K, Blaney NT, Feaster DJ, Baldewicz T, Burkhalter JE, Leeds B (1999). A randomized controlled clinical trial of a bereavement support group intervention in human immunodeficiency virus type 1-seropositive and -seronegative homosexual men. Arch Gen Psychiatry.

[29] Weiss JJ, Mulder CL, Antoni MH, de Vroome EM, Garssen B, Goodkin K (2003). Effects of a supportive-expressive group intervention on long-term psychosocial adjustment in HIV-infected gay men. Psychother Psychosom.

[30] Millard T, Agius PA, McDonald K, Slavin S, Girdler S, Elliott JH (2016). The positive outlook study: a randomised controlled trial evaluating online self-management for HIV positive gay men. AIDS Behav.

[31] Gayner B, Esplen MJ, DeRoche P, Wong J, Bishop S, Kavanagh L (2012). A randomized controlled trial of mindfulness-based stress reduction to manage affective symptoms and improve quality of life in gay men living with HIV. J Behav Med.

[32] Khumsaen N, Stephenson R (2019). Feasibility and acceptability of an HIV/AIDS self-management education program for HIV-positive men who have sex with men in Thailand. AIDS Educ Prev.

[33] Zhang P, Gao J, Wang Y, Sun Q, Sun X (2019). Effect of chronic disease self-management program on the quality of life of HIV-infected men who have sex with men: An empirical study in Shanghai, China. Int J Health Plann Manag.

[34] Li J, Mo PKH, Kahler CW, Lau JTF (2021). A three-arm randomised controlled trial to evaluate the efficacy of a positive psychology and social networking intervention in promoting mental health among HIV-infected men who have sex with men in China. Epidemiol Psychiatr Sci.

[35] Webel AR (2010). Testing a peer-based symptom management intervention for women living with HIV/AIDS. AIDS Care.

[36] Cuca YP, Asher A, Okonsky J, Kaihura A, Dawson-Rose C, Webel A (2017). HIV stigma and social capital in women living with HIV. J Assoc Nurses AIDS Care.

[37] Spies G, Konkiewitz EC, Seedat S (2018). Incidence and persistence of depression among women living with and without HIV in South Africa: a longitudinal study. AIDS Behav.

[38] Bracke P, van de Straat V, Missinne S (2014). Education, mental health, and education-labor market misfit. J Health Soc Behav.

[39] Kurspahić Mujčić A, Mujčić A (2019). The relationship between education and self-reported mental and physical health. Med Glas (Zenica).

[40] Hu Y, Zhong XN, Peng B, Zhang Y, Liang H, Dai JH (2019). Comparison of depression and anxiety between HIV-negative men who have sex with men and women (MSMW) and men who have sex with men only (MSMO): a cross-sectional study in Western China. BMJ Open.

[41] Benjamin CL, Puleo CM, Settipani CA, Brodman DM, Edmunds JM, Cummings CM (2011). History of cognitive-behavioral therapy in youth. Child Adolesc Psychiatr Clin N Am.

[42] Rachman S (2015). The evolution of behaviour therapy and cognitive behaviour therapy. Behav Res Ther.

[43] Crepaz N, Passin WF, Herbst JH, Rama SM, Malow RM, Purcell DW (2008). Meta-analysis of cognitive-behavioral interventions on HIV-positive persons' mental health and immune functioning. Health Psychol.

[44] Gellatly J, Bower P, Hennessy S, Richards D, Gilbody S, Lovell K (2007). What makes self-help interventions effective in the management of depressive symptoms? Meta-analysis and meta-regression. Psychol Med.

[45] Newby JM, Twomey C, Yuan Li SS, Andrews G (2016). Transdiagnostic computerised cognitive behavioural therapy for depression and anxiety: a systematic review and meta-analysis. J Affect Disord.

[46] Asrat B, Schneider M, Ambaw F, Lund C (2020). Effectiveness of psychological treatments for depressive symptoms among people living with HIV/AIDS in low- and middle-income countries: a systematic review and meta-analysis. J Affect Disord.

[47] Drozd F, Skeie LG, Kraft P, Kvale D (2014). A web-based intervention trial for depressive symptoms and subjective well-being in patients with chronic HIV infection. AIDS Care.

[48] Heckman TG, Heckman BD, Anderson T, Lovejoy TI, Markowitz JC, Shen Y (2017). Tele-interpersonal psychotherapy acutely reduces depressive symptoms in depressed HIV-infected rural persons: a randomized clinical trial. Behav Med.

[49] Heckman TG, Markowitz JC, Heckman BD, Woldu H, Anderson T, Lovejoy TI (2018). A randomized clinical trial showing persisting reductions in depressive symptoms in HIV-infected rural adults following brief telephone-administered interpersonal psychotherapy. Ann Behav Med.

[50] van Luenen S, Garnefski N, Spinhoven P, Kraaij V (2018). Guided internet-based intervention for people with HIV and depressive symptoms: a randomised controlled trial in the Netherlands. Lancet HIV.

[51] Thiruchselvam T, Patel A, Daros AR, Jain E, Asadi S, Laposa JM (2020). A multidimensional investigation of anxiety sensitivity and depression outcomes in cognitive-behavioral group therapy. Psychiatry Res.

[52] Okumura Y, Ichikura K (2014). Efficacy and acceptability of group cognitive behavioral therapy for depression: a systematic review and meta-analysis. J Affect Disord.

[53] Cuijpers P, Berking M, Andersson G, Quigley L, Kleiboer A, Dobson KS (2013). A meta-analysis of cognitive-behavioural therapy for adult depression, alone and in comparison with other treatments. Can J Psychiatr.

[54] Cuijpers P, van Straten A, Bohlmeijer E, Hollon SD, Andersson G (2010). The effects of psychotherapy for adult depression are overestimated: a meta-analysis of study quality and effect size. Psychol Med.

[55] Meuldijk D, Carlier IV, van Vliet IM, van Veen T, Wolterbeek R, van Hemert AM (2016). The clinical effectiveness of concise cognitive behavioral therapy with or without pharmacotherapy for depressive and anxiety disorders; a pragmatic randomized controlled equivalence trial in clinical practice. Contemp Clin Trials.

