# Supplementary material for: The benefits of psychosocial interventions for mental health in men who have sex with men living with HIV: a systematic review and meta-analysis
Source: BMC Psychiatry. 2022 Jun 29;22:440. doi: 10.1186/s12888-022-04072-1 (PMC9241196; doi:10.1186/s12888-022-04072-1)
Supplement: Supplementary file 2 — Additional file 2. Detailed search strategy. [file 12888_2022_4072_MOESM2_ESM.docx]

**Additional file 2.Detailed search strategy.**

| **Database** | **Index and keyword terms** |
| --- | --- |
| **PubMed** | hiv [mesh] OR acquired immunodeficiency syndrome [mesh] OR hiv infections [mesh] OR hiv [tiab] OR hiv infection* [tiab] OR aids [tiab] OR acquired immunodeficiency syndrome [tiab]  AND  psychotherapy [mesh] OR mental health services [mesh] OR self-care [mesh] OR self-help groups [mesh] OR telemedicine [mesh] OR therapy, computer-assisted [mesh] OR psychosocial intervention [mesh] OR psychotherap* [tiab] OR psychological therap* [tiab] OR psychological treatment* [tiab] OR psychological intervention* [tiab] OR psychosocial intervention* [tiab] OR counsel* [tiab] OR cbt [tiab] OR cognitive behavioral therapy [tiab] OR behavior therap* [tiab] OR behaviour therap* [tiab] OR interpersonal therap* [tiab] OR coping [tiab] OR peer support [tiab] OR social support [tiab] OR problem solving [tiab] OR stress manage* [tiab] OR self-help [tiab] OR internet therap* [tiab] OR online therap* [tiab] OR psychoed* [tiab] OR training [tiab] OR exposure [tiab] OR relaxation [tiab] OR mindfulness [tiab] OR reinforcement [tiab] OR risk reduction [tiab] OR commitment therap* [tiab] OR case manage* [tiab]  AND  (homosexuality, male [mesh] OR homosexuality, male[tiab] OR men who have sex with men [tiab] OR MSM [tiab] OR gay [tiab] OR bisexual men [tiab])  Filters used controlled clinical trial or randomized controlled trial, publication date from 1996/01/01. |
| **EMBASE** | 'acquired immune deficiency syndrome'/exp OR 'human immunodeficiency virus'/exp OR 'human immunodeficiency virus infection'/exp OR hiv:ab,ti OR ’hiv infection*':ab,ti OR aids:ab,ti OR 'acquired immunodeficiency syndrome':ab,ti  AND  'psychotherapy'/exp OR 'mental health service'/exp OR 'self care'/exp OR 'telemedicine'/exp OR 'self help'/exp OR 'computer assisted therapy'/exp OR 'psychosocial intervention'/exp OR psychotherap*:ab,ti OR 'psychological therap*':ab,ti OR 'psychological treatment*':ab,ti OR 'psychological intervention*':ab,ti OR 'psychosocial intervention*':ab,ti OR counsel*:ab,ti OR cbt:ab,ti OR 'cognitive behavioral therapy':ab,ti OR 'behavior therap*':ab,ti OR 'behaviour therap*':ab,ti OR 'interpersonal therap*':ab,ti OR coping:ab,ti OR 'peer support':ab,ti OR 'social support':ab,ti OR 'problem solving':ab,ti OR 'stress manage*':ab,ti OR 'self help':ab,ti OR 'internet therap*':ab,ti OR 'online therap*':ab,ti OR psychoed*:ab,ti OR training:ab,ti OR exposure:ab,ti OR relaxation:ab,ti OR mindfulness:ab,ti OR reinforcement:ab,ti OR 'risk reduction':ab,ti OR 'commitment therap*':ab,ti  AND  'male homosexuality'/exp OR 'homosexuality, male':ab,ti OR 'men who have sex with men':ab,ti OR msm:ab,ti OR gay:ab,ti OR bisexual men:ab,ti  Filters used randomized controlled trial or controlled clinical trial, publication year 1996 –2021. |
| **Cochrane Library** | "HIV Infections"[Mesh] OR "HIV"[Mesh] OR "Acquired Immunodeficiency Syndrome"[Mesh] OR (HIV):ti,ab,kw OR (HIV infection*):ti,ab,kw OR (AIDS):ti,ab,kw OR (acquired immunodeficiency syndrome):ti,ab,kw  AND  "Psychotherapy"[Mesh] OR "Mental Health Services"[Mesh] OR "Self Care"[Mesh] OR "Self-Help Groups"[Mesh] OR "Telemedicine" [Mesh] OR "Therapy, Computer-Assisted" [Mesh] OR "Psychosocial Intervention" [Mesh] OR (psychotherap*):ti,ab,kw OR (psychological therap*):ti,ab,kw OR (psychological treatment*):ti,ab,kw OR (psychological intervention*):ti,ab,kw OR (psychosocial intervention*):ti,ab,kw OR  (counsel*):ti,ab,kw OR (cbt):ti,ab,kw OR (cognitive behavioral therapy):ti,ab,kw OR (behavior therap*):ti,ab,kw OR (behaviour therap*):ti,ab,kw OR (interpersonal therap*):ti,ab,kw OR (coping):ti,ab,kw OR (peer support):ti,ab,kw OR (social support):ti,ab,kw OR (problem solving):ti,ab,kw OR stress manage*):ti,ab,kw OR (self-help):ti,ab,kw OR (internet therap*):ti,ab,kw OR (online therap*):ti,ab,kw OR (psychoed*):ti,ab,kw OR (training):ti,ab,kw OR (exposure):ti,ab,kw OR (relaxation):ti,ab,kw OR (mindfulness):ti,ab,kw OR (reinforcement):ti,ab,kw OR (risk reduction):ti,ab,kw OR (commitment therap*):ti,ab,kw OR (case manage*):ti,ab,kw  AND  "Therapy, Computer-Assisted"[Mesh] OR "Internet"[Mesh] OR "Computers"[Mesh] "Mobile Applications"[Mesh] OR Computer*[Title/Abstract] OR internet[Title/Abstract] OR web[Title/Abstract] OR web-based[Title/Abstract] OR technology[Title/Abstract] OR application[Title/Abstract] OR apps[Title/Abstract] OR e-health[Title/Abstract] OR m-health[Title/Abstract] OR mhealth[Title/Abstract] OR teleconferencing[Title/Abstract] OR self-help[Title/Abstract] OR self-support[Title/Abstract] OR behaviour therapy[Title/Abstract] OR behavior therapy[Title/Abstract] OR cognitive[Title/Abstract] OR cognition[Title/Abstract] OR coping[Title/Abstract] OR self-report*[Title/Abstract] OR self-efficacy[Title/Abstract] OR self-management[Title/Abstract]  Filters used trials, publication year 1996–2021. |
| **PsycINFO** | DE (hiv OR aids OR hiv infections) OR TX (hiv OR aids OR hiv infection*)  AND  DE (psychotherapy OR psychotherapeutic techniques OR psychosocial intervention OR mental health programs OR Counseling OR Stress management OR case management OR self management OR Telemedicine OR Computer Assisted Therapy OR Psychoeducation) OR TX (psychotherap* OR psychological-therap* OR psychological-treatment OR psychological-intervention OR psychosocial intervention* OR counsel* OR cbt OR cognitive behavioral therapy OR behavio#r-therap* OR interpersonal-therap* OR coping OR peer-support OR social-support OR problem-solving OR stress-manage* OR self-help OR internet-therap* OR online-therap* OR psychoed* OR training OR exposure OR relaxation OR mindfulness OR reinforcement OR risk-reduction OR commitment-therap* OR case-manage*)  AND  DE (homosexuality, male) OR TX (homosexuality, male OR men who have sex with men OR MSM OR gay)  Filters used publication year 1996–2021, experimental replication or treatment outcome/clinical trial or follow-up study. |
