# Supplementary material for: The benefits of psychosocial interventions for mental health in men who have sex with men living with HIV: a systematic review and meta-analysis
Source: BMC Psychiatry. 2022 Jun 29;22:440. doi: 10.1186/s12888-022-04072-1 (PMC9241196; doi:10.1186/s12888-022-04072-1)
Supplement: Supplementary file 3 — Additional file 3. Detailed characteristics in selected studies. [file 12888_2022_4072_MOESM3_ESM.docx]

**Additional file 3. Detailed characteristics in selected studies**

**Table****1** **Detailed characteristics in selected studies (N=12)**

| **Author**  **(Year)** | **Intervention** | **Control group** | **Outcomes and measures** | **Follow-up**  **(months)** | **Therapists** | **Dropout**  **(%)** | **ITT** |
| --- | --- | --- | --- | --- | --- | --- | --- |
| Antoni et al  (2006) | Group CBSM and MAT  –10 weekly sessions and each lasted for 135 min | Placebo | Depression by BDI  Anxiety by POMS-A | 3, 9, and 15 | Postdoctoral fellows and psychology graduate students | 22 | Yes |
| Blashill et al  (2017) | Individual CBT-BISC  –12weekly sessions for 50 min each | TAU | Depression by MADRS | 6 | clinical psychologist | 11 | Yes |
| Brown et al  (2019) | Group coping effectiveness training and stress management  –two sessions and each lasted for 4 h | Waiting list | Stress by The 10-item Perceived Stress Scale  Social support by SPS  Self-efficacy by The 15-item coping self-efficacy  Questionnaire | 3 | MSM facilitators | 9 | Yes |
| Carrico et al  (2005) | Group CBSM  –10 weekly sessions and each lasted for 135 min | Waiting list | Depression by BDI  Social support by SPS | 6 | Two advanced clinical-health psychology graduate students | 66 | Yes |

*(table continued)*

**Table 1 (*continued)***

| **Author**  **(Year)** | **Intervention** | **Control group** | **Outcomes and measures** | **Follow-up**  **(months)** | **Therapists** | **Dropout**  **(%)** | **ITT** |
| --- | --- | --- | --- | --- | --- | --- | --- |
| Chesney et al  (2003) | Group CET  –10 weekly sessions for 90 min each | Placebo | Depression by CESD  Anxiety by STAI  Stress by the Perceived Stress Scale  Social support by the Social Relationships Scale  Self-efficacy by a 26-item measure of perceived self-efficacy. | 6 and 12 | Leaders with graduate experience in social work and psychology or HIV services | 33 | NO |
| Goodkin et al  (1999) | Group Bereavement support intervention   –10 weekly sessions for 90 min each | TAU | Depression by HRSD  Anxiety by HARS | _ | Psychotherapists | 0 | NO |
| Weiss et al  (2003) | Supportive-expressive group intervention  –17 weekly sessions and each lasted for 2.5 h | Placebo | Depression by BDI  Anxiety by POMS-A  Social support by SSQ-6 | 15 | Psychotherapists | 14 | Yes |
| Millard et al  (2016) | Group online self-management  –7 weekly sessions and each lasted for 90min | TAU | Social support by HeiQ-SIS  Self-efficacy by POSE | 3 | Computer | 39 | Yes |

*(table continued)*

**Table 1 (*continued)***

| **Author**  **(Year)** | **Intervention** | **Control group** | **Outcomes and measures** | **Follow-up**  **(months)** | **Therapists** | **Dropout**  **(%)** | **ITT** |
| --- | --- | --- | --- | --- | --- | --- | --- |
| Gayner et al  (2012 ) | Group MBSR  –8 weekly sessions and each lasted for 3h | TAU | Depression by HADS-depression  Anxiety by HADS-anxiety  Stress by IES | 6 | Psychotherapists | 18 | Yes |
| Khumsaen et al  (2019) | Individual and group HASMEP  –14 weekly sessions for 120 to 150 min | TAU | Quality of life by WHOQOL  Social support by the HIV self-management scale- Social support | _ | Trained-peer leaders | 0 | NR |
| Zhang et al  (2019) | Group CDSM  –12 monthly sessions, NR | TAU | Quality of life by MOS-HIV scale scores -Quality of life | 12 | Psychologist | 15 | Yes |
| Li et al  (2021) | Group TGT-SN  –everyday for a month, NR | Placebo | Depression by CESD  Anxiety by GAD  Social support by the two item measure of Social support | 3, 6 and 12 | Computer | 14 | No |

Note. ITT = Intention to treat Analysis; CBSM = Cognitive behavioral stress management; MAT = Medication adherence training; CBT-BISC = Cognitive behavioral therapy for body image and self-care; CET = coping effectiveness training; MBSR = Mindfulness-based stress reduction; HASMEP = HIV/AIDS self-management education program; CDSM = Chronic disease self-management; TGT-SN = Three Good Things with electronic social networking; NR = Not reported in paper; BDI = Beck Depression Inventory; POMS-A = Profile of Mood States-Anxiety; SPS = Social Provisions Scale; MADRS = Montgomery-Asberg Depression Rating Scale; CESD = Center for Epidemiologic Studies-Depression Scale; STAI = State-Trait Anxiety Inventory; HRSD = Hamilton Rating Scale for Depression; HARS = Hamilton Anxiety Rating Scale; SSQ-6 = the 6-item version of the Social Support Questionnaire; HeiQ-SIS = Outcomes of health education-Social integration and support; POSE=the positive outlook self-efficacy scale; IES = The Impact of Event Scale; WHOQOL = World Health Organization Quality of Life; MOS-HIV = Medical Outcomes Study HIV Health Survey; GAD = The seven-item General Anxiety Disorder Scale.
