# Supplementary material for: The benefits of psychosocial interventions for mental health in men who have sex with men living with HIV: a systematic review and meta-analysis
Source: BMC Psychiatry. 2022 Jun 29;22:440. doi: 10.1186/s12888-022-04072-1 (PMC9241196; doi:10.1186/s12888-022-04072-1)
Supplement: Supplementary file 4 — Additional file 4. Quality of included studies. [file 12888_2022_4072_MOESM4_ESM.docx]

**
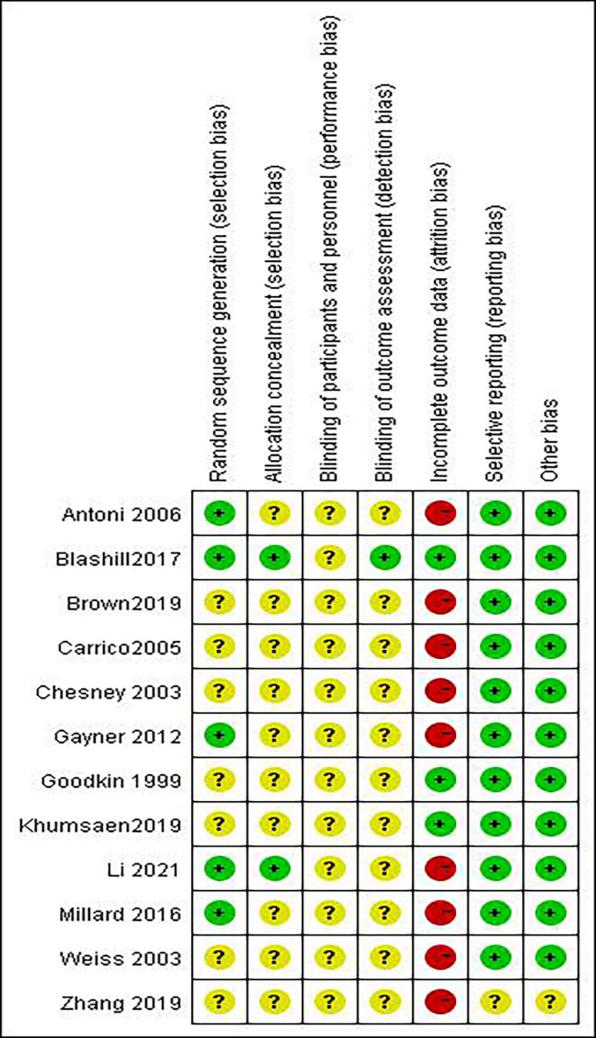
Additional file 4. Quality of included studies**

**eFigure 1. Risk of bias’ summary**

**
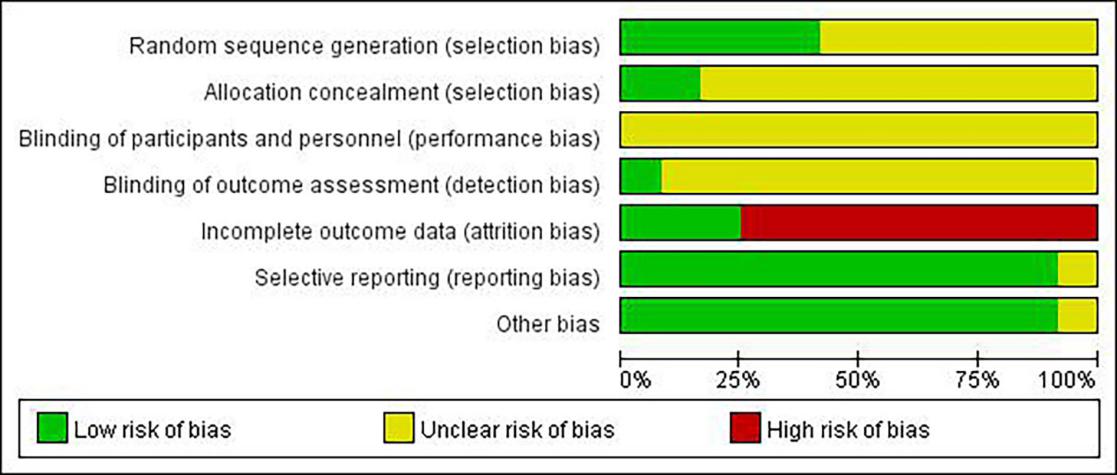
**

**eFigure 2. Risk of bias graph**
