# Supplementary material for: The benefits of psychosocial interventions for mental health in men who have sex with men living with HIV: a systematic review and meta-analysis
Source: BMC Psychiatry. 2022 Jun 29;22:440. doi: 10.1186/s12888-022-04072-1 (PMC9241196; doi:10.1186/s12888-022-04072-1)
Supplement: Supplementary file 5 — Additional file 5. Forest plot of effect sizes for anxiety, stress and social support. [file 12888_2022_4072_MOESM5_ESM.docx]

**Additional file 5.** **Forest plot of effect sizes for anxiety, stress and social support**


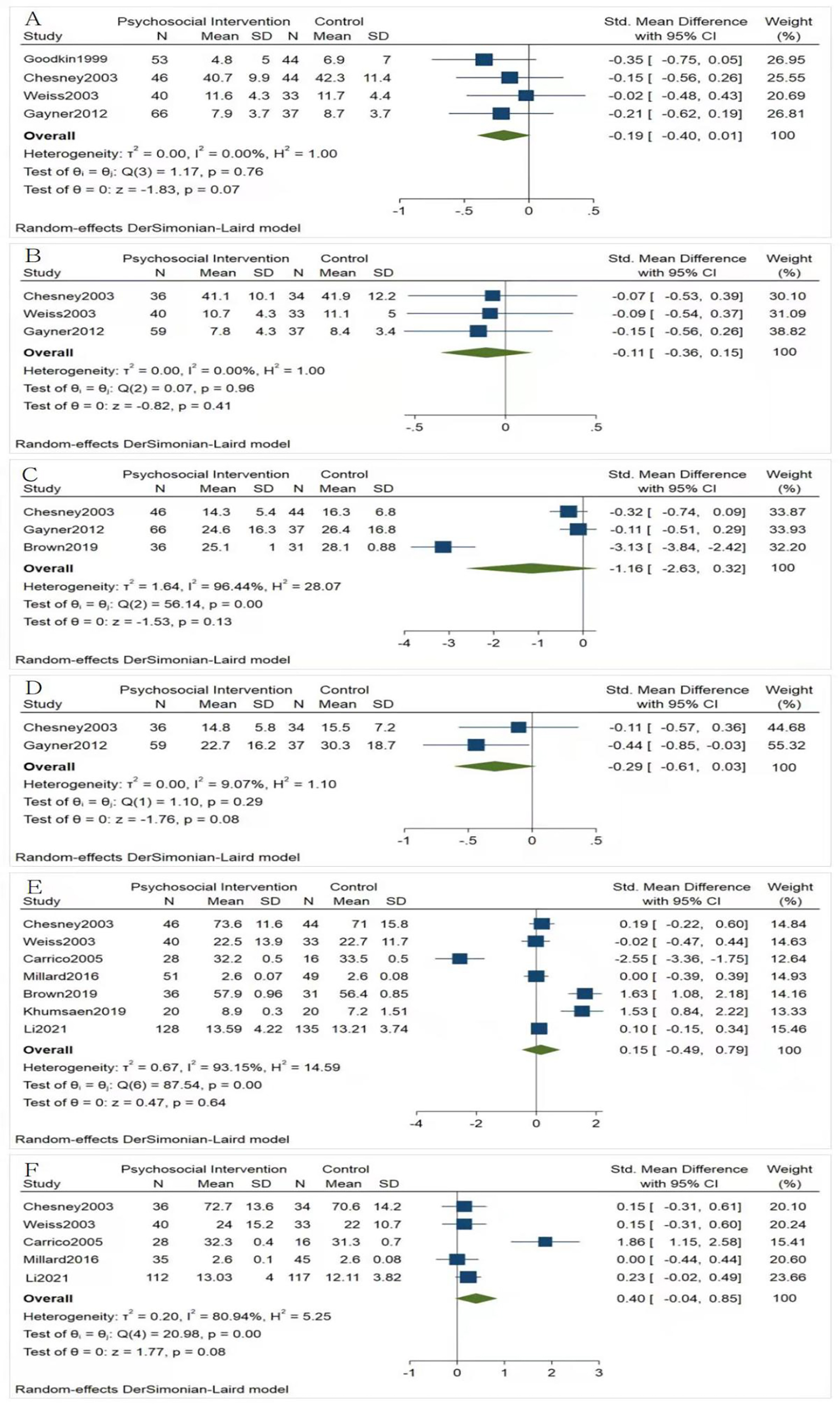


Forest plot of effect sizes for anxiety, stress and social support

A: Forest plot of effect sizes for anxiety measure at post intervention (n = 4);

B: Forest plot of effect sizes for anxiety measure at follow-up (n = 3);

C: Forest plot of effect sizes for stress measure at post intervention (n = 3);

D: Forest plot of effect sizes for stress measure at follow-up (n = 2);

E: Forest plot of effect sizes for social support measure at post intervention (n = 7);

F: Forest plot of effect sizes for social support measure at follow-up (n = 5);
